# Review

**Published:** 1908-04-11

**Authors:** 


### Review.

The Licensed Trade: An Independent Survey. Pp. vii. and 329.

Licensing and Temperance in Sweden, Norway, and Denmark. By Edwin A. Pratt. (London: Jno. Murray, Albemarle Street. 1907. Pp. x and 111.)

Mr. Pratt has long taken an active interest in various social and economic questions, and in these two volumes he gives us the views he has formed on the licensing problem as this is to be studied in Great Britain and in Scandinavia and Denmark. The larger book is written, "from the point of view of the actual traders, as seen, however, by an independent investigator." In history and in experience Mr. Pratt finds a defence and justification of the moderate use of alcoholic beverages rather than of the practice of total abstinence. He has, of course, nothing but condemnation for drunkenness, and for this he anticipates that increasing education will prove a sufficient remedy. In not a few instances he makes effective comment on the exaggerated statements and conclusions of some of the more extreme representatives of the teetotal party, and altogether his book is excellent reading, even for those who view the subject from a different standpoint from that occupied by the author. The questions associated with methods of licensing and with the amount of control that should be exercised by the State or the municipality over the drink traffic, are not particularly appropriate to our columns, but we say that Mr. Pratt brings to their discussion very full information, and for the strong views which he professes he has facts and arguments which are at least worth attention.

